# A new species of Chaetaglaea (Lepidoptera, Noctuidae, Noctuinae, Xylenini), from eastern North America

**DOI:** 10.3897/zookeys.558.6853

**Published:** 2016-02-01

**Authors:** Ken H. Stead, Jim T. Troubridge

**Affiliations:** 1 Box 168 Scotland, ON N0E 1R0; 2 23396 Mullins Ave, Port Charlotte, FL 33954

**Keywords:** Chaetaglaea tremula, Chaetaglaea rhonda, dunes, taxonomy

## Abstract

*Chaetaglaea tremula* (Harvey) occurs through the Gulf States, from southern Florida, west to eastern Texas. Coastal populations, previously referred to *Chaetaglaea tremula* occurring from the Carolinas, at least as far north as Massachusetts and shoreline dunes in southwestern Ontario are recognized as distinct and described here as *Chaetaglaea rhonda*. Adults and genitalia are illustrated for *Chaetaglaea rhonda* and *Chaetaglaea tremula*.

## Introduction

Since the early 1990s, the senior author has been studying the Lepidoptera of Pinery Provincial Park and adjacent areas of Lambton County, Ontario. As a result of this work, many undescribed species and new species for Canada, were found. One such species is *Chaetaglaea rhonda* sp. n., which is associated with sand cherry (*Prunus pumila* L.) on Lake Huron’s foreshore dunes in Lambton County. This species was first discovered in Lambton County, by Ken Stead and Dr. Kirk Zufelt in 1993 and remains the only site at which *Chaetaglaea rhonda* is known to occur in Canada. At the time of its discovery in Canada, it was identified as *Chaetaglaea tremula*; however, although specimens from Florida, Louisiana, and Mississippi submitted to BOLD for DNA analysis are *Chaetaglaea tremula* (TL “Texas”), those from Ontario, coastal North Carolina, and coastal Massachusetts represent an undescribed sister species to *Chaetaglaea tremula*. In order that a name be available for biodiversity inventories and conservation work, we describe this new species as *Chaetaglaea rhonda*.

## Materials and methods

Procedures for the dissection and preparation of genitalia and terminology for genital structures and wing markings follow that of Lafontaine (2004). Molecular variation was assessed based on the 658 bp ‘barcode’ region of the first subunit of the cytochrome oxidase (*cox1*) gene (Hebert et al. 2003). DNA was extracted from one leg removed from a dried specimen, sent to and processed at the University of Guelph through the Barcode of Life Data systems (BOLD; www.lepbarcoding.org). DNA extraction, amplification and sequencing protocols for the Barcode of Life initiative are detailed in Hebert et al. (2003). Molecular sequence data were compared with phylograms constructed using the neighbour-joining method, and distance calculations were performed using the Kimura 2-parameter (K2P) distance model as implemented on the BOLD website.

## Systematics

### Chaetaglaea rhonda sp. n.

Taxon classificationAnimaliaLepidopteraNoctuidae

http://zoobank.org/4E3DF077-C472-476A-A6F3-863A8580448F

Figs 1–3 , 11 , 13

#### Diagnosis.

*Chaetaglaea rhonda* is closely related to *Chaetaglaea tremula* (Figs 4–10). There appears to be very little individual variation within populations of *Chaetaglaea rhonda*. At the type locality in Ontario, all specimens have a gunmetal gray forewing with concolourous subterminal band and dark gray-brown hindwing. However, significant variation is present between populations. In coastal North Carolina, specimens of Chaetaglaea rhonda are slightly larger (forewing length 21–22 mm vs. 18–19 mm) and the forewing is brick red with a slightly darker, browner hindwing (Fig. 3) than populations in Ontario. Among the reddish North Carolina specimens examined there appears to be little variation. *Chaetaglaea tremula* exhibits tremendous individual variation (Figs 4–10) with the forewing varying from brick red to tan, brown, or black. The subterminal area of the forewing of *Chaetaglaea tremula* can be concolourous with the ground colour of the forewing or much paler. Due to this variation it is difficult to provide external characters that reliably separate the species; however, in *Chaetaglaea tremula*, the anal margin of the forewing normally has a pale beige line, bordered by a brick red fringe. In *Chaetaglaea rhonda*, the ground colour of the forewing extends to the posterior margin, which is bordered by a distinct red fringe. Internally, in *Chaetaglaea tremula* the costal margin of the valve is produced dorsally to form a short, subapical pointed ridge (see Fig. 12c). In *Chaetaglaea rhonda* this subapical ridge is absent. The dorsal processes of the sacculus are asymmetrical in both species, but are noticeably longer in *Chaetaglaea rhonda* (Fig. 11a) than *Chaetaglaea tremula* (Fig. 12a). The female genitalia of *Chaetaglaea rhonda* are roughly similar to those of *Chaetaglaea tremula*; however, the distal sclerotized section of the ductus bursae of *Chaetaglaea rhonda* (Fig. 13) is slightly longer and narrower than that of *Chaetaglaea tremula* (Fig. 14). DNA sequence data are congruent with the morphological data, showing that of the 658 COI base pairs examined, there is a 2.44% difference between *Chaetaglaea tremula* (Florida, Louisiana, and Mississippi) and *Chaetaglaea rhonda* (Ontario and North Carolina).

**Figures 1–10.**
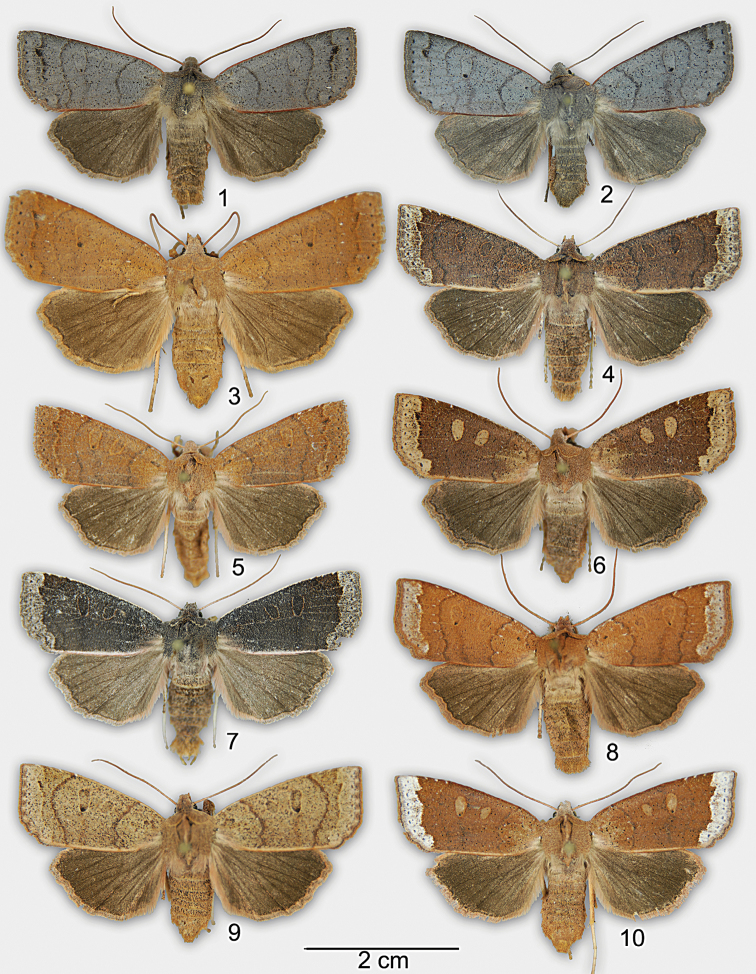
Adults of *Chaetaglaea rhonda* and *Chaetaglaea tremula*. **1** *Chaetaglaea rhonda*, male, Port Franks, ON **2** *Chaetaglaea rhonda*, female, Port Franks, ON **3** *Chaetaglaea rhonda*, female, Carolina Beach State Park, NC **4–10** *Chaetaglaea tremula*, Ocala National Forest, FL.

**Figures 11–12.**
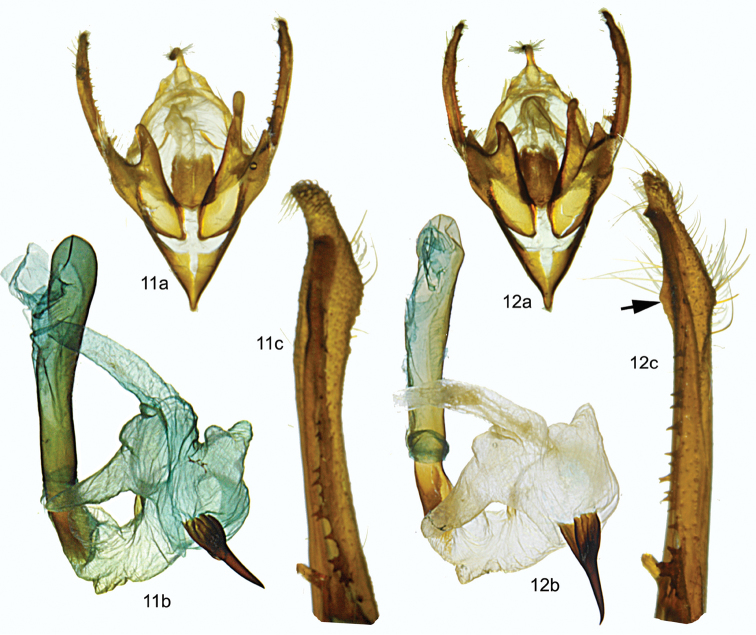
Male gentalia of *Chaetaglaea rhonda* and *Chaetaglaea tremula*. **11** *Chaetaglaea rhonda*: **a** armature **b** aedeagus with vesica everted **c** tip of right valve **12** *Chaetaglaea tremula*: **a** armature **b** aedeagus with vesica everted **c** tip of right valve; arrow shows subapical ridge absent in *Chaetaglaea rhonda*.

**Figures 13–14.**
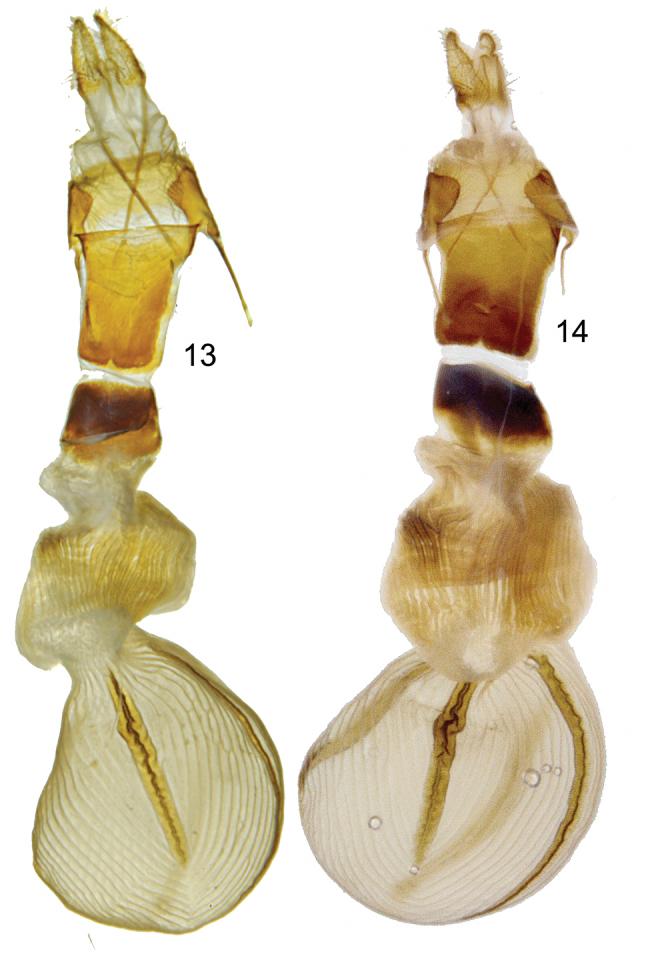
Female gentalia of *Chaetaglaea rhonda* and *Chaetaglaea tremula*. **13** *Chaetaglaea rhonda* **14** *Chaetaglaea tremula*.

#### Description.

Males and females similar. **Holotype.** Antennae filiform, ciliate; palpi red; head, vertex, prothoracic collar, thorax, and abdomen gray. Forewing length 19mm. **Dorsal forewing** glossy gunmetal gray (brick red in specimens from the Atlantic coast) with numerous black scales, costal and posterior margins red; darker gray antemedial and postmedial lines evenly concave from costa to vein CuA2, where both lines turn slightly toward outer margin; subterminal line slightly lighter gray than ground colour, scalloped between veins below vein M3; between veins M3 and R5 line evenly convex, terminating closest to outer margin on vein R5 and then bending inward toward costa. Black scales occur along anterior margin of subterminal line forming a distinct black spot in cell M5; these black scales gradually fade between vein M5 and costa and below vein M1. Outer margin with a series of black dots between veins; orbicular and reniform spots poorly demarcated by thin gray lines; a black dot occurs in lower margin of reniform spot; fringe gray. **Dorsal hindwing** dark gray brown with concolourous fringe. **Male genitalia.** (Fig. 11) Uncus narrow, terminating in fine hook. Juxta more-or-less rectangular with concave dorsal margin. Sacculus with ventral triangular process and elongate, finger-like dorsal process, left process much longer than right. Valve excavated above sacculus, leaving elongate, narrow arm terminating in setose, bubble-like, membranous cucullus. Heavily sclerotized costal margin with row of medial teeth terminates in short, subapical claw. Ampulla of clasper finger-like, extends dorsally above costa of valve. Vesica bends ventrally to the right with small sclerite on dorso-anterior margin of bend and large, posterior, bulbous, thorn-like cornutus. Two small sub-basal diverticulae on right extend to anterior and posterior; apical diverticulum terminates in ductus seminalis, above which a globular subapical diverticulum on right splits into elongate dorsal and posterior arms; a bulbous diverticulum occurs on left and a short, bi-lobed diverticulum arises from ventral surface. **Female genitalia.** (Fig. 13) Ovipositor lobes pointed with scattered setae; sclerotized plates occur on ventral and dorsal surfaces of ductus bursae between ostium bursae and a 0.5mm unsclerotized section, after which a second pair of sclerotized plates occur anterior to appendix bursae; appendix bursae with a thickened, almost leathery wall, the surface of which is very lightly sclerotized on the ventral side; ductus seminalis arises on ventral surface of appendix bursae at corpus bursae; corpus bursae egg shaped with prominent ridges and four elongate signa.

#### Type material.

**Holotype** male: Canada: Ontario, Port Franks, [Lambton County], 43.226 N 81.923 W, 17 ix 2015, J. Troubridge and K. Stead, in the Canadian National Collection of Insects, Arachnids, and Nematodes, Ottawa, Canada. **Paratypes**: 13 males, 11 females: same data as holotype.

#### Etymology.

We take pleasure in naming this species to honour Rhonda Landry, who gave support and encouragement to the authors by providing us with good food, coffee and conversation. It is a noun in apposition.

#### Distribution.

In Canada, this species is presently known only from dunes along the shore of Lake Huron in Lambton County, Ontario. In the United States, we have examined specimens from Carolina Beach State Park, New Hanover County, North Carolina. Additional specimens of *Chaetaglaea rhonda* have been submitted to BOLD for DNA analysis from the Frances A. Crane Wildlife Management Area, Barnstable County, Massachusetts, and Pine Knoll Shores, Carteret County, North Carolina. Each of these US sites are close to the Atlantic beaches and we expect that *Chaetaglaea rhonda* occurs in suitable habitats up and down the Atlantic seaboard.

## Supplementary Material

XML Treatment for Chaetaglaea rhonda
